# Phytosynthesized Metallic Nanoparticles—between Nanomedicine and Toxicology. A Brief Review of 2019′s Findings

**DOI:** 10.3390/ma13030574

**Published:** 2020-01-25

**Authors:** Irina Fierascu, Ioana Catalina Fierascu, Roxana Ioana Brazdis, Anda Maria Baroi, Toma Fistos, Radu Claudiu Fierascu

**Affiliations:** 1 National Institute for Research & Development in Chemistry and Petrochemistry—ICECHIM Bucharest, Emerging Nanotechnologies Group, 202 Spl. Independentei, 060021 Bucharest, Romania; irina.fierascu@icechim.ro (I.F.); roxana.brazdis@icechim.ro (R.I.B.); anda.baroi@icechim.ro (A.M.B.); toma.fistos@icechim.ro (T.F.); 2 University of Medicine and Pharmacy “Carol Davila”, 37 Dionisie Lupu Str., 030167 Bucharest, Romania; 3 Zentiva Romania S.A., 50 Theodor Pallady Blvd., 032266 Bucharest, Romania

**Keywords:** phytosynthesized nanoparticles, antimicrobial, antitumoral, toxicology

## Abstract

Phytosynthesized nanoparticles represent a continuously increasing field of research, with numerous studies published each year. However, with the emerging interest in this area, the quality of the published works is also continuously increasing, switching from routine antioxidant or antimicrobial studies on trivial microbial lines to antibiotic-resistant strains or antitumoral studies. However, this increasing interest has not been not reflected in the studies regarding the toxicological effects of nanoparticles (NPs); this should be a subject of greatest interest, as the increasing administration of NPs in general (and phytosynthesized NPs in particular) could lead to their accumulation in the environment (soil, water and living organisms). The present review aims to present the most recent findings in the application of phytosynthesized NPs as antimicrobial and antitumoral agents, as well as the results regarding their toxicological potential.

## 1. Introduction

**Figure 1:**
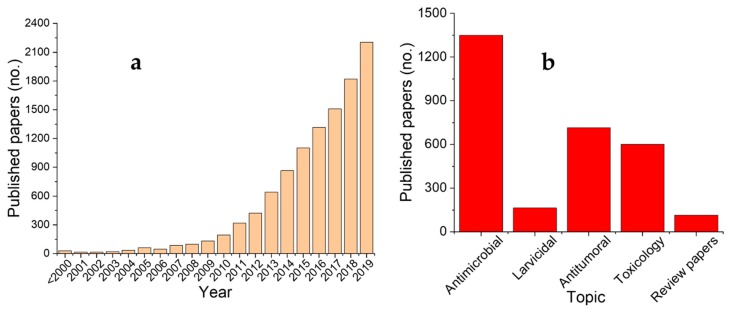
Published papers on the topic of the current review. (**a**) Studies on the general topic of nanoparticles phytosynthesis and (**b**) studies on the selected topics, both published in 2019. The relatively high number of papers published on the topic “toxicity” was due to the overlapping of other applications (antimicrobial and antitumoral) and due to keywords/abstract description provided by the authors.

In the last few decades, nanotechnology has offered a series of valuable tools for applications in different areas, ranging from antimicrobial coatings, cosmetics or wound dressing to fabrics and clothing or detergents [1]. Their increased use in such various applications could lead to their accumulation in the environment, which could cause potentially adverse effects both on the environment and to human health [2]. However, recent advances in the field of nanoparticle phytosynthesis have led to their proposal as potential agents in multiple biomedical applications, offering a viable alternative to the use of environmentally hazardous reagents (in the case of traditional chemical synthesis) or expensive equipment (necessary in physical methods), as previously presented by our group [3]. Another major advantage of the phyto approach is represented by the wide variety of vegetal materials (with very different composition in terms of phytoconstituents involved in the nanoparticles synthesis process) that are available to produce nanoparticles with hierarchical structures [3]. In spite the numerous advantages of this approach, the process still has some bottle-necks, the most important of which is represented by the difficulties in obtaining homogenous nanoparticles in terms of shape and, especially, size, as well as the elucidation of the specificity of individual biomolecules [3]. Though several types of metallic nanoparticles that are synthesized by using plant extracts have been studied in terms of their toxic potential towards target organisms (leading to their proposal as antimicrobial [4], antiviral [5] or cytogenotoxic agents [6]), their possible negative toxicity has not been thoroughly established. The present review aims to present the advances that have been recorded in the last year in the area of phytosynthesized nanoparticle applications and toxicity studies, as well to underline the shortcomings of the encountered approach. The literature review survey was performed on multiple databases (Scopus, Web of Science, ScienceDirect, SpringerLink, PubMed) and used the main keywords “nanoparticles” and “extract” (as the term “phytosynthesis” was not adopted by all the authors). From the returned results, only the works that were published in the last year (2019) were selected. Furthermore, a supplementary selection was performed by using keywords as “antimicrobial,” “cytotoxicity,” and “toxicology” (results presented in Figure 1). The results of this preliminary selection led to the return of approximately 1800 unique works (from all the databases). A preliminary validation was manually performed (by reading the keywords and abstract) in order to remove the “false-positive” results. The final validation was performed by reading the entire manuscript. The criteria for inclusion in the present review were: (i) the use of phytosynthesized nanoparticles that were obtained in the laboratory; (ii) the existence of analytical studies for the characterization of the nanoparticles; and (iii) in-depth applications reports. The review is structured in different chapters for each potential application that is related to the toxic effect of the metallic nanoparticles.

Figure 1a reveals the increasing interest in the area of nanoparticle phytosynthesis. Most of the articles dealing with their synthesis and potential applications cover their antioxidant properties (not subject of the present review), followed by antimicrobial properties. Additionally, several studies cover multiple applications, thus explaining the difference between the sum of papers presented in Figure 1b and the total number of papers identified, as previously mentioned.

## 2. Antimicrobial Applications

**Figure 2:**
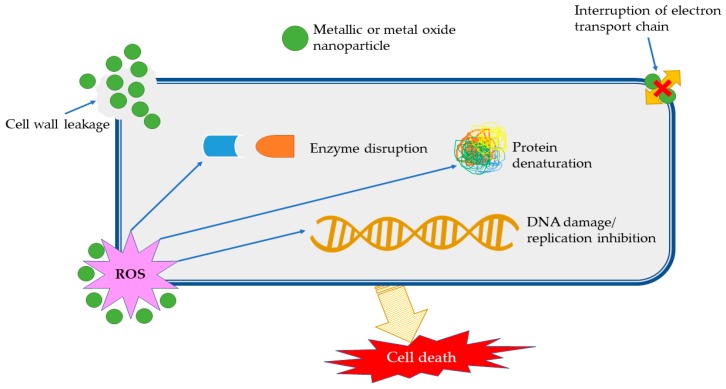
Antimicrobial mechanism of nanoparticles (adapted from [9,10,11,12]).

As previously stated, the vast majority of the published literature covers the antimicrobial application of phytosynthesized nanoparticles. The antimicrobial mechanism of the nanoparticles has been thoroughly established [7] and has mainly been based on the disruption of cellular membrane functionality and the generation of reactive oxygen species (ROS); see Figure 2. The cellular internalization of the nanoparticles is specific for each type of nanoparticle (including phagocytosis, pinocytosis and passive penetration), with the entry and cytosolic access into cells being influenced by a series of factors such as their shape, size, functionalization, surface charge, or protein corona [8].

As expected, due to their traditional antimicrobial use [13], silver nanoparticles (NPs) are the most encountered nanoparticles that have been proposed in phytosynthesis studies for antimicrobial application. Table 1 presents some relevant studies regarding the antimicrobial potential of phytosynthesized silver nanoparticles.

**Figure 3:**
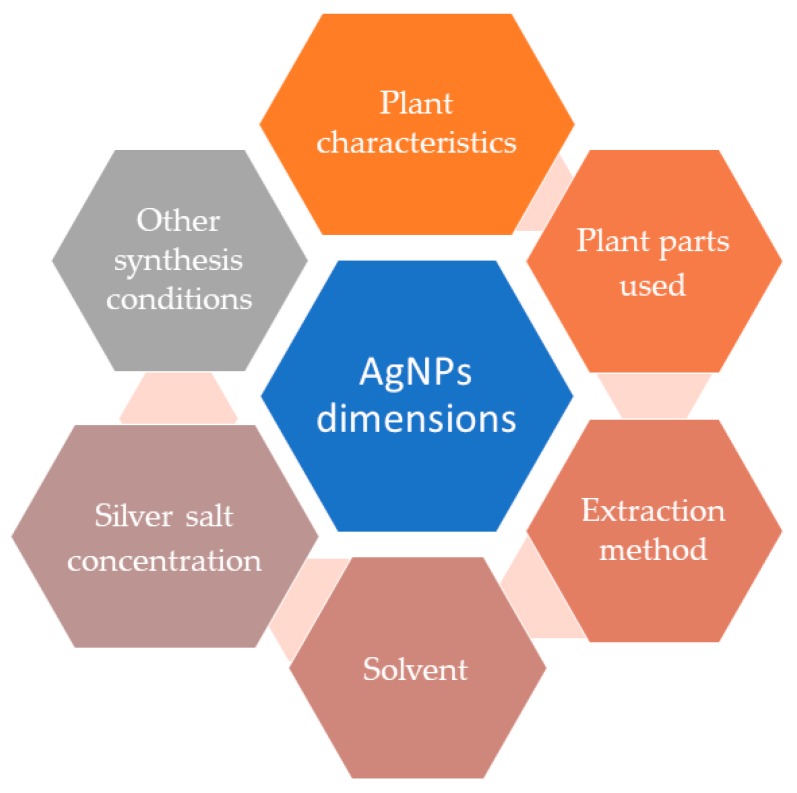
Factors influencing the morphological characteristics of silver nanoparticles and, consequently, their antimicrobial properties.

The studies presented in Table 1 proposed the phytosynthesis of silver nanoparticles by using different plant materials (bark, rhizomes, flowers, etc.) and different solvents (alcohols, acetone, water) or extraction procedures (classical temperature extraction, Soxhlet, microwave or ultrasound-assisted) (the factors influencing the NPs morphology and their antimicrobial properties are presented in Figure 3). It can be noticed that the large majority of the studies presented the synthesis of spherical NPs with dimensions under 50 nm. Though the dimensions were similar and the lines used in the antimicrobial studies were common, significant differences could be observed between the obtained results (expressed either as minimum inhibitory concentration or inhibition zone diameter). Due to the similarities in terms of size and shape, the most probable explanation for the registered differences resides in the capping phytochemicals from different extracts (with a superior antimicrobial effect being associated with an increased content in polyphenolic compounds).

For example, Mtambo et al. [15] presented the phytosynthesis of silver nanoparticles (AgNPs) by using aqueous extracts obtained from different parts of *Bidens pilosa* L. 1753 (leaves, stems and roots), as well as different concentration of a metal salt precursor (1 and 2 mM). The lowest average diameters that were observed by the authors were 7.85 nm (leaf extract/1 mM silver salt) and 11.89 nm (root extract/2 mM silver salt). Thus, the authors emphasized the importance of not only the plant part used for the extraction but also of the silver salt precursor concentration. Generally speaking, the higher the concentration of the silver salt, the the larger obtained nanoparticles. The antimicrobial properties, studied against a series of Gram-positive and Gram-negative bacteria, revealed a concentration-dependent effect (over the concentration range of 6.25–200 mg/L), with a correlation between the observed dimensions and the antibacterial effect only for the leaf extract. The root extracts had the weakest antibacterial potential, although the NP dimensions were close to those of the leaf extract. This could have been caused by the lower polyphenolic content (compared with the stem extract), and by their contribution to the total antibacterial effect. The antifungal potential was also established to be concentration-dependent, with a superior effect for the smaller particles (obtained by using 1 mM silver precursor) lines and the best efficiency observed for the leaf extract, followed by stem and root extracts. Ibrahim et al. [16] obtained spherical and cubical nanoparticles with larger dimensions (30–90 nm) by using African juniper leaf acetone extracts. Their antimicrobial assay (performed by measuring the inhibition zone in well-diffusion assay) revealed a superior effect of the NPs compared to the positive control (penicillin; 10 μg) against all lines (180%–350%), an effect to which the crude extract also contributes (with inhibition zones that were comparable to the positive control). Another interesting study (especially because of the number of the bacterial lines it used) is represented by the work of Zangeneh et al. [45]. By using *Stachys lavandulifolia* flower aqueous extracts that were obtained by using classical temperature extraction, the authors obtained nanoparticles that were mostly spherical with 20–40 nm diameters. They also evaluated the antimicrobial potential by using a well-diffusion assay (registering inhibition zones) and through the determination of minimum inhibitory (bactericidal) concentrations (MIC/MBC). The best results were obtained for the Gram-positive lines (MIC = MBC = 2 mg/mL for *Staphylococcus saprophyticus, Bacillus subtilis* and *Enterococcus faecalis*); the same value for the MIC/MBC was obtained in the case of Gram-negative lines, but only for *Pseudomonas aeruginosa.* The weakest effect was observed for *Salmonella typhimurium, Shigella flexneri* and *Proteus mirabilis* lines (MIC = 8 mg/mL), as well as *Proteus mirabilis* (MBC = 16 mg/mL). The superior effect on Gram-positive bacteria can be mainly assigned to the absence of the outer membrane for Gram-positive bacteria (compensated by the thicker yet permeable peptidoglycan layer of the NPs [48,49].

As a general conclusion, for the same extract and phytosynthesis procedure, the best antimicrobial properties are exhibited by lower dimension nanoparticles, with a superior effect on the Gram-positive bacteria. A variation of the extract that is used for phytosynthesis also induces a variation of the obtained nanoparticle characteristics and their antimicrobial properties (dependent on the extract composition).

Several other works published in 2019 presented the antimicrobial potential of other types of phytosynthesized nanoparticles (as detailed in Table 2).

Though not as encountered in antimicrobial applications, gold nanoparticles (Au NPs) were obtained and presented in the cited studies. Among them, the study of Gopinath et al. [51] presented the potential use of Au NPs against a widely encountered gastric pathogen (*Helicobacter pylori*). The nanoparticles were phytosynthesized by using a *Tribulus terrestris* L. fruit aqueous extract and different metallic salt concentrations (1 and 2 mM). The resulting nanoparticles had dimensions of 7 nm (for 1 mM Au salt) and 55 nm (for 2 mM Au salt), with spherical and some triangular (especially for the larger NPs) morphologies. The nanoparticles proved to be efficient against several multi-drug resistant *H. pylori* strains in a concentration-dependent manner, with the larger nanoparticles being more effective. In our opinion, the effect could have been influenced by the presence of multiple morphologies in the larger particles case. Other, more exotic metallic nanoparticles (Ti NPs and Se NPs) were obtained by Seydi et al. [57] and Gunti et al. [58], respectively, by using aqueous extracts that were obtained from different plants. In both cases, the NPs were spherical, with dimensions of 22 and 15–40 nm, respectively, and they were effective against several Gram-positive bacteria, Gram-negative bacteria, and fungal lines, with MBC/MFC values close to the positive controls (commercial antimicrobials) for Ti NPs [57].

Several metal oxide nanoparticles have been presented in the literature, with ZnO NPs being the most encountered. These nanoparticles have a much more varying morphology (hexagonal, spherical, triangular, rod-shaped, etc.) and dimensions usually around 25–30 nm (although larger NPs have been recorded). They have been found to exhibit antimicrobial potential towards several Gram-positive bacteria, Gram-negative bacteria, and fungal lines in concentration-dependent manner (Table 2). For example, the hexagonal and triangular ZnO NPs (20–30 nm) that were obtained by Iqbal et al. [63] exhibited antimicrobial potential against *Escherichia coli, Staphylococcus aureus, Bacillus subtilis, Klebsiella pneumonia, Pseudomonas aeruginosa, Candida albicans, Mucor racemosus, Aspergillus niger, Fusarium solani* and *Aspergillus flavus*, with superior potential against Gram-positive bacteria (MIC = 7.8 mg/L) compared with Gram-negative bacteria (MIC = 15.625–62.5 mg/L) and fungal lines (MIC = 15.625–125 mg/L). MgO NPs were obtained in both studies, with each citing a flower-shaped morphology and different dimensions; they were also found to be active against Gram-positive and Gram-negative bacteria. Similar activities were recorded for Fe_2_O_3_ and Fe_3_O_4_ NPs [68,69], although it was observed that, for iron oxide nanoparticles, the dendrimer morphology seemed to enhance the antimicrobial potential [63]. Some rarer nanoparticles were presented by Sabouri et al. [72] and Iqbal et al. [73], who obtained NiO and CoO NPs by using natural extracts for phytosynthesis followed by a calcination step (400 and 500 °C, respectively), in order to obtain crystalline NPs. The materials showed antimicrobial potential towards Gram-positive *S. aureus* (NiO) [72], as well as Gram-positive bacteria, Gram-negative bacteria, and fungal lines (CoO, with MIC values ranging from 21.875 and 175 mg/L) [73]. A particular case was represented by bi-metallic nanoparticles. If we are speaking of doping oxides with phytosynthesized NPs or the direct phytosynthesis of bi-metallic NPs, the approach can harvest the properties of both components. This was proven by Adebayo et al. [77] and Heydari et al. [79], who evaluated bi-metallic NPs via comparisons with separate NPs. Bi-metallic Au/Ag NPs have been proven to be efficient (in some cases even superior to Ag NPs) against a series of microbial and isolates lines. Their observation is surprising, as it would be expected bi-metallic NPs to show intermediate antimicrobial properties [80]. Similarly, Fe_3_O_4_/Cu NPs have been proven to retain both their magnetic properties (from the Fe_3_O_4_ component) and antimicrobial properties (from the Cu NPs), with a minimum inhibitory (bactericidal) concentration (MIC/MBC) close to the ones obtained for Cu NPs against Gram-positive and Gram-negative bacteria [79]. This approach is particularly interesting for applications that request two different properties for the composites (such as magnetic and antimicrobial properties).

Though usually not as effective (in terms of antimicrobial properties) as the previously presented Ag NPs, the other types of nanoparticles present a wider range of morphologies, which could, in some cases, provide supplementary properties to the NPs.

## 3. Antitumoral Applications

**Figure 4:**
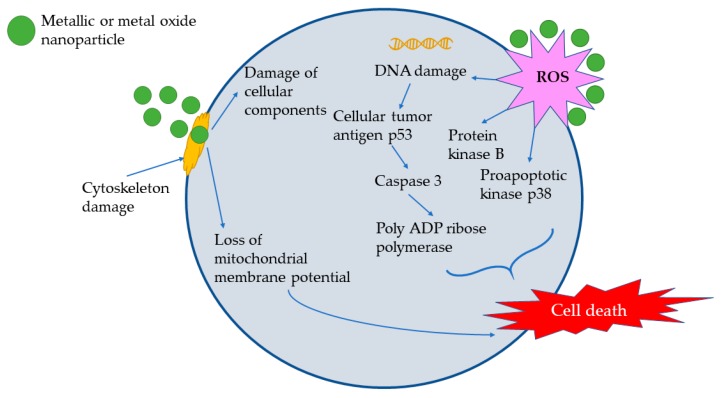
Antitumoral mechanism of nanoparticles (adapted from [73,81]).

Another important application of the toxic potential of NPs towards selected cells is represented by their antitumoral applications, usually evaluated in cytotoxicity studies against tumoral cellular lines. The cytotoxicity mechanism is similar to the antimicrobial one (Figure 4); it is mainly based on interactions with the cellular cytoskeleton, the generation of reactive oxygen species, intracellular glutathione (GSH) depletion, and damages to other cellular components (lysosomes, proteins, and mitochondria), all leading to apoptosis or cellular damage and, finally, cell death [73,81]. Again, silver and gold NPs represent the main types of nanoparticles that have been studied in this area (Table 3).

As can be seen from Table 3, several authors discussed the antitumoral properties of the NPs in studies that also covered their antimicrobial properties. From the articles that exclusively presenti the antitumoral effect, Shaniba et al. [89] described the phytosynthesis of spherical silver nanoparticles (24 nm average size) and their antitumoral evaluation against several tumoral cell lines (colorectal cancer, cervical epithelioid carcinoma, and lung carcinoma cells) by using an MTT (3-(4,5-dimethylthiazol-2-yl)-2,5-diphenyltetrazolium bromide) assay, fluorescence, and scanning electron microscopy on cells that were stained with different dyes (propidium iodide, acridine orange/ethidium bromide, annexin) in order to visualize apoptosis signs. By using the MTT assay, the authors identified a dose-dependent cell inhibition. At the same time, the NPs inhibited cell migratory abilities, induced cell cycle arrest, and mitochondria-mediated apoptosis. Similar observations were made by Karuppaiya et al. [90], who used phytosynthesized silver nanoparticles against breast and gastric human cancer cell lines. Their results suggested a superior effect on the gastric cancer cells, the antiproliferative effect being mediated by nuclear condensation or DNA fragmentation. Vinay et al. [92] used both cell lines and the *Allium cepa* assay for the evaluation of the cytotoxicity of phytosynthesized NPs, identifying antimitotic activity and chromosomal aberrations (chromosome-breaks, chromosome-stickiness, laggard chromosomes, and clumped chromosomes) that represented very good indicators of the genotoxic potential, thus making them a viable alternative for replacing cell lines in antitumoral studies [93]. Gold nanoparticles that were obtained by using green tea extract were also proved to possess antitumoral effects on different cell lines (human gastric adenocarcinoma, epithelial cervix adenocarcinoma, hepatocyte carcinoma and colorectal adenocarcinoma cells) [96]. More importantly, the authors compared the effect of phytosynthesized Au NPs with different sizes and shapes (spheres and stars), with the rod-shaped being obtained via chemical synthesis. As proven by their results, the size had little impact on the cytotoxicity properties compared with the morphology. Thus, the best results were obtained for the rod-shaped NPs (not presented in Table 3, EC_50_ (half maximal effective concentration) = 22.7 μM), followed by the star-shaped and spherical NPs, although their dimensions increased in the order of sphere to rods to stars [96].

Besides the silver and gold nanoparticles, several other metallic, metal oxides, metal sulfides and bi-metallic nanoparticles have also been presented as possessing effective antitumoral properties against several tumoral cells (Table 4).

Among the published works, some less encountered materials that possess a high antitumoral potential have been presented. This is the case, for example for the phytosynthesized Se NPs that were obtained by Krishnan et al. [116]; these NPs exhibited a 50% inhibition of cell viability of the human hepatocyte carcinoma cells (HepG2) at a 30 μg/mL concentration, and CoO NPs did the same with a half maximal effective concentration of 31.4 μg/mL against the same cell line [73].

Regarding the antitumoral studies, we must underline the fact that the relatively limited number of cell lines and protocols that have been used in these studies allowed for the pertinent comparison of the results, thus representing a good starting-point for future studies. At the same time, most of the proposed NPs seemed to have a good efficiency against the various tumoral cells, with half maximal effective concentrations usually under 100 mg/L. For example, against breast adenocarcinoma cells (MCF-7), the best results in the MTT assay were achieved by using spherical silver nanoparticles that were obtained by using *Tropaeolum majus* L. leaf extracts (EC_50_ = 2.49 mg/L) [24], followed by spherical gold nanoparticles that were obtained by using *Anacardium occidentale* L. leaves (EC_50_ = 6 mg/L) [53]; the same types of NPs were also found to be efficient against human cervical epithelioid carcinoma cells (HeLa) (Ag NPs that were obtained by using *Piper longum* leaves—EC_50_ = 5.27 mg/L [88] and Au NPs that were obtained by using *Alternanthera sessilis* leaves—EC_50_ = 10 mg/L [99]), while the most effective nanoparticles against human lung carcinoma cells (A549) were proven to be silver NPs that were obtained by using *Scorzonera calyculata* aerial parts (EC_50_ = 12.5 mg/L) [35], gold NPs that were obtained by using *Marsdenia tenacissima* leaves (EC_50_ = 15 mg/L) [95], MgO NPs that were obtained by using *Sargassum wightii* (EC_50_ = 37.5 mg/L) [70], and ZnO NPs that were obtained by using *Allium cepa* leaves (EC_50_ = 51.25 mg/L) [66].

## 4. Toxicological and Biocompatibility Studies

Multiple studies regarding the antitumoral potential of the NPs also presented the biocompatibility of the materials, as evaluated by using normal cell lines (Table 5). For example, on monkey kidney epithelial cells (VERO) nanoparticles were found to be toxic, with the EC_50_ value varying from under 10 mg/L (Ag NPs) to 30 mg/L (ZnO NPs) or even over 150 mg/L (Fe NPs). On human embryonic kidney cells (HEK-293), most of the studies presented EC_50_ values in the range of hundreds or thousands of mg/L (for ZnO, Au and Ag NPs). The same observation can be made for all the normal cell lines presented in Table 5.

From the presented studies, it can be concluded that the phytosynthesized nanoparticles present a very good specificity towards tumoral cells as compared to normal cells, supporting their potential antitumoral use, as well as their good safety in general applications, or in case of accidental contact with human organism.

**Figure 5:**
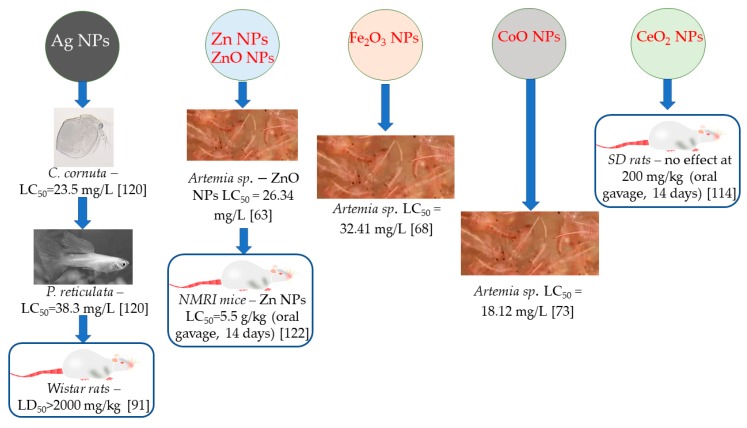
Relevant findings of the in vivo toxicological studies using phytosynthesized nanoparticles; rodent studies are highlighted.

The toxicological studies regarding the phytosynthesized nanoparticles represent the major bottle-neck in the current approach. A few studies have presented their effect on wild life, generally in studies performed on aquatic organisms (especially brine shrimps, *Daphniidae, Cyclopidae* or *Paramecium* sp.) and, rarely, on other animals (Table 6 and Figure 5).

Among the presented studies, Jenifer et al. [120] comparatively studied the toxicity of silver nanoparticles and silver ions on invertebrate and vertebrate aquatic animals. By using spherical Ag NPs with dimensions between 10 and 50 nm, they studied their effects on the water flea (*Ceriodaphnia cornuta*)*,* unicellular ciliates (*Paramecium* sp.), and guppy fish (*Poecilia reticulata*). Their results suggested toxic effects on the invertebrates (LC_50_ = 23.5/15.5 mg/L, 100% lethality at 50/30 mg/L after 24 h/5 min, while the lower concentration of toxic led to abnormal swimming behavior and morphological abnormalities), with higher limits in vertebrates (LC_50_ = 38.3/34.5 mg/L after 48/96 h, 100% mortality at 50 mg/L after 96 h, the increase concentration leading to a heart rate decrease). However, those toxic effects were lower than those observed for silver ions (from silver nitrate), both for invertebrates (100% mortality at 30 and 10 mg/L, respectively) and vertebrates (100% mortality after 48 h exposure to 40 mg/L), with morphological and physiological abnormalities recorded at lower concentrations (5 mg/L and lower heart rates at similar concentrations in fishes). Their conclusions were that the NPs can interact with the aquatic animals’ cell membranes, thus leading to disruption in the membrane potential that is associated with the ion-efflux disturbance. The study is of particular importance as the accumulation/effect on the planktonic organisms could affect the entire aquatic ecosystem, as those organisms represent the primary producers in those systems [120]. The findings of Odeyemi et al. [91] regarding the toxicity of Ag NPs on rats could be correlated with the work of El-Maksoud et al. [123], who demonstrated that chemically-obtained AgNPs at 50 mg/kg body weight (b.w.) exhibited hepatotoxicity in rats (severe hydropic degeneration and inflammatory cell infiltration in the portal area, focal hepatic necrosis, the degeneration of the biliary, the epithelium of the bile duct, the congestion of the portal vein, and the proliferation of the fibroblast) [123].

In terms of phytotoxicity, silver NPs (spherical, 15 nm, obtained by using the aqueous extract of *Veronica officinalis* L.) were proven to be non-toxic (in the concentration range of 0.0009–0.0675 mg/mL) towards *Linum flavum* and *Lepidium sativum* seeds [124], while the application of magnetite NPs (semi-spherical, 29.8 nm, obtained by using a *Fumaria officinalis* L. aqueous extract) led to growth reduction and significant changes in the total phenol, total flavonoid content, and antioxidant enzymes’ activity of the aquatic plant *Azolla filiculoides* (at 0.5–10 mg/L concentrations) [125].

## 5. Recent Findings in the Morphology-Properties Correlation

The correlation between nanoparticles’ morphology and their antimicrobial or anti-tumoral activities was the subject of several valuable published works in the last few years. For example, the antibacterial effect of nanoparticles has previously been presented to be superior in the case of smaller dimension NPs in studies against different bacterial and fungal lines [126,127,128]. At the same time, spherical nanoparticles have been shown to possess a superior antimicrobial potential compared with cubical, plate-shaped or triangular nanoparticles [126,129].

This general rule also applies for phytosynthesized nanoparticles (as presented in Table 1 and Table 2). However, due to the influence of the natural extract (exhibited both as a reaction matrix and as the phytoconstituents coating the nanoparticle), the literature has offered examples regarding the superior antimicrobial effect of larger nanoparticles. For example, in the case of similar silver nanoparticle morphologies (spherical), Subramanian et al. [27] recorded a minimum inhibitory concentration (MIC) of 2.5 mg/L (against *S. aureus*) and 0.5 mg/L (against *E. coli*) for 22.7 nm NPs, while Dakshayani et al. [20] recorded MIC values of 25 mg/L against both lines when using 5–10 nm NPs. The difference in antimicrobial efficiency, assigned to the used extract, could be exploited in future studies that have focused on the most effective plants and extraction procedures for obtaining phytosynthesized NPs with enhanced antimicrobial activity. The same discussion is also valid for the influence of NPs shape. Though spherical NPs are considered to be the most effective antimicrobial nanoparticles, nanoparticles with heterogenous morphologies [28] have been proven to have superior antimicrobial potentials compared to spherical NPs [32] with approximatively the same size.

These examples are provided only to underline the fact that a comparison between the results of different studies (using different plants or even different extraction techniques) can prove to be misleading. A thorough comparison between the effects of different sizes and shapes on the final properties usually requires the same characteristics of the natural extract used for phytosynthesis. Tanase et al. [34] evaluated the antimicrobial potential of different sized Ag NPs (tuned by varying the synthesis pH) against *S. aureus,* methicillin-resistant *S. aureus, E. coli, K. pneumoniae,* and *P. aeruginosa.* In all cases, the the MIC and MBC (minimum bactericidal concentration) were significantly lower for the smaller dimension NPs. Very interestingly, the results of Gopinath et al. [51] on different sized Au NPs revealed that larger Au NPs (55 nm) proved more efficient (although with small differences; statistical significance not presented by the authors) against multiple multi-drug resistant *H. pylori* strains. As previously stated, this could be explained by the presence of different shaped NPs (not only spherical), although the exact mechanism (as also presented by the authors) remains to be elucidated.

The same previously discussed morphological characteristics affect the anti-tumoral potential of nanoparticles. Literature data suggest that nanospheres possess the weakest cytotoxic potential (in the case of Ag and Au NPs), with the most promising morphologies being the nanowires (Ag NPs) [130] and the nanostars (Au NPs) [131]. El-Hawary et al. [84] studied the potential of Ag NPs that were obtained by using two cultivars (with similar compositions) of *Jasminum sambac* L. The nanoparticles with smaller dimensions (8.83 nm) exhibited a higher cytotoxic potential against MCF-7 cells and human bladder carcinoma cells (5637) (Table 3) and lower toxicity towards immortal keratinocyte cells (HaCaT) (Table 4), as compared with the higher dimension NPs. When evaluating the overall influence of the morphology of the NPs, literature data suggest that the shape represents a more important factor than the size [96]. Thus, a comparison of phytosynthesized Au NPs spheres (8.7 nm) and stars (99 nm) with chemically obtained nanorods (length/width = 60.4/16.4 nm) revealed a superior effect of nanostars (IC_50_ = 81.8 μM, compared with the nanospheres—IC_50_ = 127.1 μM), although both were inferior to the nanorods (IC_50_ = 22.7 μM) against human hepatocyte carcinoma cells (HepG2). As corroborated with their findings regarding the cellular uptake of the NPs (best for nanospheres—58%—followed by nanorods and nanostars), the results support the conclusion that the final cytotoxic potential of the NPs represents the results of synergic influence of multiple factors [96].

The variation of the antimicrobial and cytotoxic potential of the phytosynthesized nanoparticles (in comparison with the NPs that are obtained by using a radiation-assisted approach) was recently presented by our group [132] and supported the previously presented conclusion. Thus, although the phytosynthesized NPs had larger dimensions, their antimicrobial potential was higher (enhanced for the phytosynthesized NPs with lower dimensions). The radiation-assisted NPs were proven, in turn, to possess a superior cytotoxic potential (which was also enhanced with the NPs’ decrease in diameter).

In order to correctly define the influence of the various factors on the different potential of the NPs, studies that evaluate the variation of the final properties with each factor in similar phytosynthesis procedures are necessary.

## 6. Concluding Remarks and Perspectives

As previously presented, the number of articles about metallic nanoparticles phytosynthesis is increasing from year to year. This could be explained not only by the overall increase of the published scientific literature but also by a growing interest in this area. The field of phytosynthesized NPs, will, in our opinion, continuously grow in the following years, as the use of different plant extracts and metallic salts precursors can offer a tremendous variety of differently shaped and sized nanoparticles. At the same time, the thorough understanding and a successful control of the phytosynthesis process in general towards homogenous nanoparticles could benefit from further studies; the continuous search for new alternatives to chemically or physically synthesized nanoparticles for various applications could find an adequate response in this area.

Phytosynthesized NPs are close to industrial use for human-related applications. Phytosynthesized Au NPs (obtained by using aqueous extracts of *Morinda lucida* Benth. leaves) of a specific size and shape (spherical and 10 nm) have been proven to be able to penetrate *Stratum Corneum* by intercellular paths, opening the possibilities to use the NPs as transdermal transporter [133].

Considering their tremendous potential, the use of phytosynthesized NPs is expected in the near future to pass the barrier from laboratory studies to clinical trials. In this this context, it is worth mentioning that in the Cochrane Database of Systematic Reviews (section Clinical Trials), the metallic or metal oxide nanoparticles represent the subject of very few trials, while the phytosynthesized nanoparticles have not yet been evaluated. For example, some nanoparticles that were obtained by chemical reduction were evaluated in clinical trials, including Ag NPs’ antimicrobial activity and skin irritation potential (30 participants) [134], the antimicrobial potential of denture tissue conditioners, including Ag and ZnO NPs (42 participants) [135], Ag NPs for the treatment of pyorrhea (25 participants) [136], Ag NP-based sprays for reducing the pain that is associated with cesarean wounds (92 participants) [137], Ag NPs as antimicrobial coatings for venous catheters (472 participants) [138]. This would suggest the possibility, in the near future, of developing clinical trials, including phytosynthesized nanoparticles.

Phytosynthesized nanoparticles represent a continuously increasing field of research, with numerous studies published each year. Together with the high interest in this area, the quality of the published works is also continuously increasing, switching from routine antioxidant or antimicrobial studies on trivial microbial lines to antibiotic-resistant strains and antitumoral studies. However, this growing interest is not reflected in the studies regarding the toxicological effects of NPs; this should be a subject of particular concern, as the increasing use of NPs in general (and the proposal of phytosynthesized NPs for future applications in particular) could lead to their accumulation in the environment.

At the same time, the focus of the researchers should be also switched towards the phytosynthesis of other metallic NPs or metal oxide NPs, as well as the evaluation of their potential applications and toxicological effects.

## Figures and Tables

**Table 1:** Antimicrobial potential of phytosynthesized silver nanoparticles (as presented in studies published in 2019).

Extract Used	NPs Characteristics	Microbial Lines	Antimicrobial Effect	Ref.
*Tecoma stans* (L.) Juss. ex Kunth flowers aq. extract	Spherical, 50–60 nm	Gram-positive (*Staphylococcus aureus*) and Gram-negative (*Escherichia coli*) bacteria.	IZ = 24/16 mm	[14]
*Bidens Pilosa* L. 1753 leaves, stem and root aq. extract	Spherical, 7.85–26.11 nm	Gram-negative (*Pseudomonas aeruginosa, Klebsiella pneumonia*, *Escherichia coli*), Gram-positive bacteria (*Enterococcus faecalis, Staphylococcus aureus*), fungi (*Candida albicans/C. krusei/C. parapsilosis*).	IE = 52%–56.6%/45.9%–56.1%/48.2%–58.4%/42.2%–46.4%/39.6%/47.4%/67.1%–88.77%/80%–88.3%/88.2%–94.7% at 200 mg/L,	[15]
*Juniperus procera* Hochst. ex Endl. leaves ac. extract	Spherical, cubic, 30–90 nm	Gram-positive (*Bacillus subtilis, Micrococcus luteus*), Gram-negative bacteria (*Proteus mirabilis, Klebsiella pneumoniae*), fungi (*Candida albicans*).	IZ = 28/28/29/18/24 mm	[16]
*Berberis vulgaris* L. leaves and root aq. extract	Spherical, 30–70 nm	Gram-positive (*S. aureus*), Gram-negative bacteria (*E. coli*).	MIC = 400/100 mg/L	[17]
*Trigonella foenum-graecum* L. aq. extract	Spherical, 33.93 nm (average diameter)	*E. coli, Klebsiella pneumoniae, S. aureus, Salmonella typhi, Pseudomonas aeruginosa, Aspergillus flavus*, *C. albicans, Trichophyton rubrum, Penicilium notatum, Trichoderma viridiae.*	MIC = 125/250/62.5/500/500/250/500/250/500/250 mg/L	[18]
*Salvia hispanica* L. seeds aq. extract	Spherical, 1–23 nm	*E. coli, S. aureus*	IZ = 18.5/15.3 mm at 7.5 μg	[19]
*Selaginella bryopteris* leaves meth.: aq. (1:1) extract	Spherical, 5–10 nm	*E. coli, S. aureus, A. niger*	MIC = 25/25/100 mg/L	[20]
*Paulownia tomentosa* (Thunb.) Steud. leaves eth., isoprop., aq. Soxhlet extract	Mainly spherical, 10–45 nm	*P. aeruginosa, S. aureus*	Visible inhibition zone (not quantified)	[21]
*Persea americana* Mill. seed aq. extract	Spherical, oblongated, 50 nm	*E. coli*	IZ = 1.5 mm at 334.11 mg/L	[22]
*Bergenia ciliate* (Haw.) Sternb. 1831, *Bergenia stracheyi* (Hook.f. and Thorns.) 1868, *Rumex dentatus* L., *Rumex hastatus* D.Don	Spherical, 25–73 nm	*S. aureus, S. haemolyticus, B. cereus, E. coli, S. typhi, P. aeruginosa*	MIC = 0.25–1 (*P. aeruginosa, S. typhi*); 0.25–0.75 (*S. aureus*); 0.25–0.5 (*E. coli*), 0.75 (*B. cereus*), 4 mg/mL (*S. haemolyticus*)	[23]
*Tropaeolum majus* L. leaves aq. Soxhlet extract	Spherical, 35–55 nm	*S. aureus*, *E. faecalis*, *E. coli, S. typhi, P. aeruginosa*, *A. niger*, *C. albicans*, *P. notatum*, *Trichoderma viridiae*, *Mucor* sp.	Inhibition of the tested strains, no quantification provided	[24]
*Coriandrum sativum* L. leaves aq. extract	Spherical, 11.9 nm	*Pasteurella multocida, Enterobacter aerogenes, S. aureus, B. subtilis*	IZ = 10/11/12/14 mm at 200 μL	[25]
*Allium sativum* L. aq. extract	Spherical, 10–50 nm	*S. aureus, P. aeruginosa*	IZ = 17.4/19.2 mm at 100 mg/L	[26]
*Oldenlandia umbellata* L. leaves aq. extract	Spherical, 22.7 nm	*Streptococcus mutans, S. aureus, E. coli, P. aeruginosa*	MIC = 1.5/2.5/0.5/1.5 mg/L	[27]
*Juniperus chinensis* L. leaves 80% meth. extract	Heterogenous morphology, 18–25 nm	*E. coli, P. aeruginosa, S. aureus, B. subtilis*	MIC = 15/14/18/17 mg/L	[28]
*Artocarpus integer* Spreng. leaves aq. extract	Spherical, 5.76–19 nm	*S. aureus, B. cereus, E. coli, Salmonella entertica*	IZ = 14/17/15/16 mm at 25 μg	[29]
*Artemisia absinthium* L. aq. extract	Pseudospherical, 2–80 nm	*C. albicans, C. parapsilosis, C. krusei*	MIC = 0.325 mg/L at a 6:4 nitrate/extract ratio	[30]
*Clinacanthus nutans* (Burm.f.) Lindau leaves meth. extract	Spherical, 77.8–85.3 nm	*B. subtilis, E. faecalis, S. aureus, E. coli, P. aeruginosa, Proteus vulgaris*	IZ = 11.5/8.33/8.67/8.5/9/8.8 mm at 10 μL	[31]
*Murraya koenigii* L. leaves aq. extract	Spheroidal, 5–20 nm	*S. aureus, E. coli*	MIC = 32/16–64 mg/L	[32]
*Myrtus communis* L. leaves aq. extract	Spherical, 5–30 nm	*S. aureus, E. coli*	MIC = 12.5/25 mg/L	[33]
*Fagus sylvatica* L. bark aq. extract	Spherical, polygonal, triangular, 32–62 nm (pH-dependent)	*S. aureus, E. coli, Klebsiella pneumoniae, P. aeruginosa*	MIC = 0.09–0.34/0.19–0.54/0.99–2.74/0.15–0.41 mg/mL (dependent on metal source)	[34]
*Scorzonera calyculata* Boiss aerial part eth.: aq. extract	Spherical, 25.28 nm	*S. aureus, Listeria monocytogenes, B. subtilis, K. pneumoniae, P. aeruginosa, S. pyogenes*	MIC = 125/62.5/125/31.25/62.5/250 mg/L	[35]
*Derris trifoliata* Lour seeds aq. extract	Spherical, 16,92 nm	*K. pneumonia, S. aureus, E. coli, P. aeruginosa*	IZ = 20/36/19.5/ absent at 0.03 mg	[36]
*Azadirachta indica* A.Juss., 1830 leaves aq. extract	Spherical, 29 nm	*Penicillium sp., Fusarium sp., Aspergillus sp. Ralstonia solanacearum*	92%/89%/69% inhibition after 6 days, respectively, MIC = 200 mg/L	[37]
*Impatiens balsamina* L., *Lantana camara* L. leaves aq. extracts	Spherical, 12–20/3.2–12 nm	*S. aureus, E. coli*	IZ = 11.03–13.8/13.9–15.8 mm (*S. aureus*), 8.9–10.2/15.4–17.7 (*E. coli*)	[38]
*Rosa santana* petals aq. extract	Spherical, 6.52–25.24 nm	*S. aureus, E. coli*	IZ = 11.73/10.20 mm	[39]
*Reinwardtia indica* Dumort. leaves eth. Soxhlet extract	Spherical, 3–15 nm	*S. aureus, E. coli, P. aeruginosa, C. albicans*	IZ = 14.2/13.6/15.9/14.1 mm	[40]
*Morus alba* L. fruits aq. extract	Spherical, 80–150 nm	*E. coli, L. monocytogenes*	IZ = 24.87/26.93 at 5%	[41]
*Uncaria gambir* Roxb. leaves aq. extract	Spherical, 6–41 nm	*S. aureus, E. coli*	IZ = 16/14 mm	[42]
*Olea europaea* L. leaves aq. extract	Spherical, 10–22 nm	*Coliforms*	Absence of colonies at 50 mg/L	[43]
*Corymbia citriodora* (Hook.) K.D. Hill and L.A.S. Johnson leaves eth. extract	Spherical, 17.51 nm	*Acinetobacter baumannii, E. coli, P. aeruginosa, K. pneumoniae, E. faecalis, S. aureus, C. albicans*	MIC_90_ = 0.04/0.04/0.04/0.04/0.04/0.09/0.02 mg/L	[44]
*Stachys lavandulifolia* flower aq. extract	Spherical 20–40 nm	*P. mirabilis, Shigella flexneri, L. monocytogenes, K. pneumonia, P. aeruginosa, E. coli, E. faecalis, B. subtilis, Streptococcus pyogenes, Staphylococcus saprophyticus, S. epidermidis, S. aureus, S. typhimurium, Streptococcus pneumonia*	IZ = 39.8–49.2 mm at 64 mg/mL	[45]
*Salvia miltiorrhiza* Bunge leaves aq. extract	Spherical, oval, hexagonal and Triangular, 12–80 nm	*S. typhi, S. flexneri, S. pyogenes, P. aeruginosa*	IZ = 10.2/10.5/10.8/9.24 at 60 μg	[46]
*Allium cepa* L. peel aq. extract	Spherical, 8–50 nm	*Bacillus sp., S. aureus, Corynebacterium sp., E. coli, Salmonella sp., Vibrio cholerae*	IZ = 17/19/17/19.3/17.7/18 mm at 100 mg/L	[47]

Where: ac.—acetone; aq.—aqueous; eth.—ethanol; IE—inhibitory effect (percentage cell death); isoprop.—isopropyl alcohol; IZ—inhibition zone; meth.—methanol; MIC—minimum inhibitory concentration; and MIC_90_—minimum inhibitory concentration required to inhibit the growth of 90% of organisms.

**Table 2:** Antimicrobial potential of other phytosynthesized nanoparticles (as presented in studies published in 2019).

NPs	Extract Used	NPs Characteristics	Microbial Lines	Antimicrobial Effect	Ref.
Au NPs	Mix of *Olea europaea* L. fruit and *Acacia nilotica* (L.) Wild. ex Delile husk aq. extracts	Spherical, with irregular forms, 44.96 nm	*Escherichia coli, Pseudomonas aeruginosa, Klebsiella pneumoniae, Staphylococcus aureus, Bacillus subtilis*	>4 mm inhibition zones, superior for Gram-negative bacteria	[50]
Au NPs	*Tribulus terrestris* L. fruit aq. extract	Spherical, few triangular, 7 nm (1 mM metal salt precursor)/55 nm (2 mM)	*Helicobacter pylori*	IZ = 10.2–12.1 mm, MIC = 16.75–21.50 mg/L	[51]
Au NPs	*Gundelia tournefortii* L. leaves aq. extract	Spherical, 40–45 nm	*C. albicans, C. glabrata, C. krusei, C. guilliermondii, P. aeruginosa, E. coli, B. subtilis, S. aureus, Salmonella typhimurium, Streptococcus pneumonia*	IZ = 33–38 mm at 64 mg/mL (against *Candida* sp.), MIC/MBC = 2–4 mg/mL	[52]
Au NPs	*Anacardium occidentale* L. leaves aq. extract	Spherical, 10–60 nm	*E. coli, B. subtilis*	IZ = 24/10 mm at 40 μL	[53]
Au NPs	*Halymenia dilatate* Zanardini aq. extract	Triangular, spherical, 16 nm	*Aeromonas hydrophila*	IZ = 21 mm at 100 mg/L	[54]
Cu NPs	*Allium saralicum* R.M. Fritsch leaves aq. extract	Spherical, 45–50 nm	*C. albicans, C. glabrata, C. krusei, C. guilliermondii, P. aeruginosa, E. coli, B. subtilis, S. aureus, S. typhimurium, S. pneumonia*	MFC = 2–8 mg/mL, MBC = 4–8 mg/mL (Gram-negative), 2–8 mg/mL (Gram-positive)	[55]
Fe NPs	*Uvaria chamae* P. Beauv. leaves aq. extract	Irregular shapes, 29.5–51.1 nm	*E. coli, P. aeruginosa, B. subtilis, S. aureus, C. albicans, A. niger*	IZ = 11/11/14/15/17/16 mm at 2 mg/mL, MIC = 0.5 mg/mL	[56]
Ti NPs	*Allium eriophyllum* Boiss leaves aq. extract	Spherical, 22 nm	*C. guilliermondii, C. krusei, C. albicans, C. glabrata, P. aeruginosa, S. typhimurium, E. coli, S. aureus, S. pneumoniae, B. subtilis*	MFC = 8–16 mg/mL, MBC = 4 mg/mL	[57]
Se NPs	*Emblica officinalis* Gaertn. fruits aq. extract	Spherical, 15–40 nm	*E. coli, Listeria monocytogenes, S. aureus, Enterococcus faecalis, A. brasiliensis, A. flavus A. oryzae, A. ochraceus, Fusarium anthophilum, Rhizopus stolonifer*	MBC = 33.17–97.5 mg/L, MFC = 10.67–38.17 mg/L	[58]
ZnO NPs	*Tecoma castanifolia* (D.Don) Melch. leaves aq. extract	Spherical, 70–75 nm	*E. coli, P. aeruginosa, S. aureus, B. subtilis*	IZ = 17/15/17/15 mm at 100 μg	[59]
ZnO NPs	*Bambusa vulgaris* Schrad. ex J.C.Wendl., *Artabotrys hexapetalus* (L. f.) Bhandari leaves aq. extracts	Spherical/spherical and rod-shaped, 15–20/20–30 nm	*Streptococcus* and *Serratia* strains	IZ = 6/5 (*Streptococcus*), 15/13 (*Serratia*) mm	[60]
ZnO NPs	*Pandanus odorifer* (Forssk.) Kuntze leaves aq. extract	Spherical, 90 nm	*B. subtilis E. coli*	IZ = 26/24 mm at 50 μg/well	[61]
ZnO NPs	*Cinnamomum tamala* (Buch.-Ham.) T. Nees and C. H. Eberm. leaves aq. extract	Spherical, hexagonal, 26.57 nm	*S. aureus*	20% inhibition at 100 mg/L	[62]
ZnO NPs	*Rhamnus virgate* Roxb. leaves aq. extract	Hexagonal, triangular, 20–30 nm	*E. coli, S. aureus, B. subtilis, K. pneumonia, P. aeruginosa, C. albicans, Mucor racemosus, A. niger, Fusarium solani, A. flavus*	MIC = 7.8–125 mg/L, best results for *S. aureus* and *B. subtilis*	[63]
ZnO NPs	*Euphorbia heterophylla* L. leaves aq. Soxhlet extract	Hexagonal, 40 nm	*S. aureus, E. coli, Pseudomonas desmolyticum, Klebsiella aerogenes*	IZ = 10.83/8.43/8.92/6.5 at 1000 mg/L	[64]
ZnO NPs	*Mentha pulegium* L. leaves aq. extract	Semi-spherical, 38–49 nm	*S. aureus, E. coli*	IZ = 22.7/19.3 at 200 mg/L	[65]
ZnO NPs	*Allium cepa* L. leaves aq. extract	Hexagonal, cubic, 50 nm	*Bacillus sp., E. coli, S. aureus, Vibrio cholerea, Corynebacterium sp., Salmonella sp.*	IZ = 20.33/20.33/20/18.33/16/17 mm at 100 mg/L	[66]
ZnO NPs	*Laurus nobilis* L. leaves aq. extract	Spherical, hexagonal, 20–30 nm	*E. coli*	MIC = 1200 mg/L	[67]
Fe_2_O_3_ NPs	*Rhamnus virgate* Roxb. leaves aq. extract	Spherical, 20 nm	*S. aureus, B. subtilis, P. aeruginosa, Klebsiella pneumoniae, E. coli, M. racemosus, A. flavus, A. niger, C. albicans, F. solani*	MIC = 31.25–125 mg/L	[68]
Fe_3_O_4_ NPs	*Artemisia haussknechtii* Boiss. leaves aq. extract	Dendrimer shaped, with branches, 1–150 nm	*E. coli, S. aureus, S. marcescens*	IZ = 12.16–13.16 at 0.1 M metallic salt precursor, MIC = 50/12.5/50 mg/L	[69]
MgO NPs	*Sargassum wightii* Greville ex J. Agardh, 1848 aq. extract	Flower shaped, 68.02 nm	*S. aureus, P. aeruginosa*	IZ = 9/8 mm at 30 mg/L, MIC = 256 mg/L, MBC = 256/1024 mg/L	[70]
MgO NPs	*Rosmarinus officinalis* L. flowers aq. extract	Flower shaped, 8.8 nm	*Xanthomonas oryzae* pv. *oryzae*	IZ = 5.1 cm at 16 mg/L	[71]
NiO NPs	*Abelmoschus esculentus* (L.) Moench leaves aq. extract	Spherical, 18.6 nm	*S. aureus, E. coli, P. aeruginosa*	IZ = 10 mm (*S. aureus*)	[72]
CoO NPs	*Geranium wallichianum* Oliv. leaves aq. extract	21 nm	*B. subtilis, S. aureus, P. aeruginosa, E. coli, K. pneumonia, M. racemosus, C. albicans, A. niger, A. flavus, F. solanai*	MIC = 21.875/87.5/175/43.75/175/21.875/43.75/21.875/175/21.875 mg/L	[73]
Ag/TiO_2_ NPs	*Acacia nilotica* (L.) Wild. ex Delile leaves aq. extract	Spherical, 17 nm	*S. aureus, E. coli, P. aeruginosa, C. albicans*	IZ = 64/64/128/64 mg/L	[74]
Au/Ag NPs	*Annona squamosa* L. aq. extract	Multiple morphologies (spherical, triangular, hexagonal, rod-shaped, etc.) 30–50 nm	*B. subtilis, S. aureus, E. coli, S. typhi*	IZ = 14.66/13.66/11/9.33 mm	[75]
Au/Ag NPs	*Piper betle* L. leaves aq. extract	Clusters, spherical	*B. subtilis, K. planticola.*	IZ = 14/13 mm at 50 μL	[76]
Au, Ag, Au/Ag NPs	*Persea americana* Mill. fruit peel aq. extract	Spherical, rod-shaped aggregates, 16–70/18–80/44–55 nm	*E. coli, B. subtilis, K. pneumoniae, L. monocytogenes, P. vulgaris, P. aeruginosa, S. aureus, S. pyogenes, A. niger, A. fumigatus, F. solani, A. flavus, C. albicans*	IE = 36%–76%/52%–94%/53%–85% at 80 mg/L	[77]
Au, ZnO and Au/ZnO core-shell NPs	*Hibiscus sabdariffa* L. leaves aq. extract	Spherical, 20–50 nm	*S. aureus*	Complete inhibition at 500/750 mg/L after 3 h	[78]
Fe_3_O_4_, Cu, Fe_3_O_4_/Cu NPs	*Carum carvi* L. seeds aq. extract	Spherical, 25/37/62 nm	*S. aureus, B. subtilis, E. coli*	MIC = 0.01/0.05/0.02 (Cu)/0.01/0.04/0.03 (composite)	[79]

Where: aq.—aqueous; Au NPs—gold nanoparticles; Cu NPs—copper nanoparticles; CoO NPs—cobalt oxide nanoparticles; CuO NPs—copper oxide nanoparticles; Fe NPs—iron nanoparticles; Fe_2_O_3_ NPs—iron(III) oxide nanoparticles; Fe_3_O_4_ NPs—iron(II, III) oxide nanoparticles; IE—inhibitory effect (percentage cell death); IZ—inhibition zone; MBC—minimum bactericidal concentration; MFC—minimum fungicidal concentration; MgO NPs—magnesium oxide nanoparticles; NiO NPs—nickel oxide nanoparticles MIC—minimum inhibitory concentration; MIC_90_—minimum inhibitory concentration required to inhibit the growth of 90% of organisms; Se NPs—selenium nanoparticles; Ti NPs—titanium nanoparticles; and ZnO NPs—zinc oxide nanoparticles.

**Table 3:** Antitumoral potential of phytosynthesized silver and gold nanoparticles (as presented in studies published in 2019).

NPs	Extract Used	NPs Characteristics	Cell Lines	Main Findings	Ref.
Ag NPs	*Bidens pilosa* L. 1753 leaves, stem and root aq. extract	Spherical, 7.85–26.11 nm	A549	MTS assay—CD = 55.6%/44.9%/43.5%	[15]
Ag NPs	*Juniperus procera* Hochst. ex Endl. leaves ac., eth. extracts	Spherical, cubic, 30–90 nm	Cow RBC	Lysis effect: 1.75%/100%	[16]
Ag NPs	*Trigonella foenum-graecum* L. aq. extract	Spherical, 33.93 nm	MCF7	MTT assay—EC_50_ = 6.25 mg/L	[18]
Ag NPs	*Tropaeolum majus* L. leaves aq. Soxhlet extract	Spherical, 35–55 nm	MCF7	MTT assay—EC_50_ = 2.49 mg/L	[24]
Ag NPs	*Allium sativum* L. aq. extract	Spherical, 10–50 nm	MCF7	MTT assay—EC_50_ = 23 mg/L	[26]
Ag NPs	*Artocarpus integer* Spreng. leaves aq. extract	Spherical, 5.76–19 nm	MCF7, MG-63	MTT assay—EC_50_ = 90/70 mg/L after 24 h	[29]
Ag NPs	*Scorzonera calyculata* Boiss aerial part eth.:aq. extract	Spherical, 25.28 nm	A549	MTT assay—EC_50_ = 12.5 mg/L	[35]
Ag NPs	*Derris trifoliata* Lour seeds aq. extract	Spherical, 16,92 nm	A549	MTT assay—EC_50_ = 86.23 mg/L after 24 h	[36]
Ag NPs	*Reinwardtia indica* Dumort. leaves eth. Soxhlet extract	Spherical, 3–15 nm	SiHa	MTT assay—CV = approx. 10% after 24 h at 500 mg/L	[40]
Ag NPs	*Olea europaea* L. leaves aq. extract	Spherical, 10–22 nm	MCF7, HeLa	CV = 48%/38% after 96 h, at 50 mg/L	[43]
Ag NPs	*Salvia miltiorrhiza* Bunge leaves aq. extract	Spherical, oval, hexagonal, triangular, 12–80 nm	LNCaP	MTT assay—CV = approx. 38% after 24 h at 100 mg/L	[46]
Ag NPs	*Allium cepa* L. peel aq. extract	Spherical, 8–50 nm	A549	MTT assay—EC_50_ = 113.25 mg/L at 24 h	[47]
Ag NPs	*Leucas aspera* (Willd.) Link leaves aq. extract	Spherical, 50 nm	HeLa	LDH assay—Ctx = 58% after 24 h at 150 mg/L	[82]
Ag NPs	*Ceiba pentandra* L. bark eth. extract	Spherical, 5–50 nm	HCT-116	MTT assay—EC_50_ = 60 mg/L	[83]
Ag NPs	*Jasminum sambac* L. (Ait) leaves eth. Extracts—two cultivars	Spherical, 8.83/11.24 nm	MCF7, 5637	EC_50_ = 6.32/17.32 (MCF7) 5.54/27.89 (5637) mg/L	[84]
Ag NPs	*Datura inoxia* Mill. flowers aq. extract	Polygonal, 15–73 nm	MCF7	MTT assay—EC_50_ = 20 mg/L after 24 h	[85]
Ag NPs	*Phoenix dactylifera* Chabaud seed eth. extract	Spherical, 17–19 nm	MCF7	MTT assay—EC_50_ = 188 mg/L	[86]
Ag NPs	*Nigella sativa* L. seeds aq. extract	Spherical, 100–150 nm	MCF7	MTT assay—EC_50_ = 10 g/L for 24 h	[87]
Ag NPs	*Piper longum* L. leaves aq. extract	Spherical, 28.8 nm	HeLa	MTT assay—EC_50_ = 5.27 mg/L after 24 h	[88]
Ag NPs	*Manilkara zapota* (L.) P. Royen leaves aq. extract	Spherical, 24 nm	HCT-116, HeLa, A549	MTT assay—EC_50_ = 8/16/29 mg/L	[89]
Ag NPs	*Dysosma pleiantha* (Hance) Woodson rhizomes aq. extract	Spherical, 76 nm	MDA-MB-231, MDA-MB-453, AGS	MTT assay—EC_50_ = 33.521/36.25/7.14 mM/L	[90]
Ag NPs	*Elaeodendron croceum* (Thunb.) DC. stem bark aq. extract	Spherical, 12.62–41.44 nm	MDA-MB-231	WST-1 method, EC_50_ = 138.8 mg/L	[91]
Ag NPs	*Rauvolfia tetraphylla* L. leaves aq. extract	Spherical, 40 nm	*Allium cepa* assay; MCF7, A549	Antimitotic activity, chromosomal aberrations; MTT assay—EC_50_ = 134.67/118.5 mg/L	[92]
Ag, Au NPs	*Aconitum toxicum* Reichenb. leaves eth., meth. extracts	Spherical, 12.22/13.45 (Au), 21.96/22.08 (Ag) nm	*Allium cepa* assay	Antimitotic activity, chromosomal aberrations	[93]
Au NPs	*Halymenia dilatate* Zanardini aq. extract	Triangular, spherical, 16 nm	HT-29	MTT assay—EC_50_ = 22.62 mg/L	[54]
Au NPs	Mix of *Olea europaea* L. fruit and *Acacia nilotica* (L.) Wild. ex Delile husk aq. extracts	Spherical, with irregular forms, 44.96 nm	MCF7, TCT-116, HCepG-2	MTT assay—EC_50_ = 45.5/37.2/40.6 μL	[50]
Au NPs	*Anacardium occidentale* L. leaves aq. extract	Spherical, 10–60 nm	MCF7	MTT assay—EC_50_ = 6 mg/L	[53]
Au NPs	*Tribulus terrestris* L. fruit aq. extract	Spherical, few triangular, 7 nm (1 mM metal salt precursor)/55 nm (2 mM)	AGS	Annexin V/Propidium Iodide staining assay CV > 70% at 24 h, for both types of NPs at 200 mg/L	[51]
Au NPs	*Lonicera japonica* L. flowers aq. extract	Spherical, triangular, hexagonal, 10–40 nm	HeLa	WST-1 method, CV = approx. 50% at 400 mg/L	[94]
Au NPs	*Marsdenia tenacissima* (Roxb.) Moon leaves aq. extract	Spherical, oval-shaped, 40–50 nm	A549	MTT assay—EC_50_ = 15 mg/L	[95]
Au NPs	*Camellia sinensis* (L.) Kuntze leaves aq. extract	Spheres, stars, 8.7/99 nm	AGS, HeLa, HepG2, HT-29	Cytotoxic towards all lines, MTT assay—EC_50_ = 127.1/81.8 μM (HepG2)	[96]
Au NPs	*Citrus macroptera* Mont. fruit juice	Pseudospherical trigonal, rod-shaped, 20 nm	A549, MDA-MB 468, HepG2	MTT assay, EC_50_ = 143/157.9/70.2 μg/L	[97]
Au NPs	*Panax notoginseng* (Burkill) F. H. Chen ex C. Y. Wu and K. M. Feng leaves aq. extract	Hexagonal, spherical, oval, triangular, 12–80 nm	PANC-1	MTT assay—CV = approx. 25% after 48 h at 30 mg/L	[98]
Au NPs	*Alternanthera sessilis* (L.) R.Br. ex DC. leaves aq. extract	Spherical, 30–50 nm	HeLa	MTT assay—EC_50_ = 10 mg/L after 24 h	[99]
Au NPs	*Eleutherococcus senticosus* (Rupr. and Maxim.) Maxim leaves and stems aq. extract	Spherical, 20 nm	B16	MTT assay—EC_50_ = 10 mg/L after 24 h	[100]
Au NPs	*Ocimum tenuiflorum* leaves aq. extract	Spherical, 2–10 nm	HeLa, MCF7, A549, H1299	MTT assay—EC_50_ = 200/~180/~220/~350 mg/L after 24 h	[101]
Au NPs	*Rabdosia rubescens* L. leaves aq. extract	Spherical, 130 nm	A549	MTT assay—EC_50_ = 50 mg/L after 24 h	[102]
Au NPs	*Dunaliella salina* (Dunal) Teodoresco aq. extract	Spherical, triangular, hexagonal, 5–45 nm	MCF7	MTT assay—CV = 20% after 48 h at 200 mg/L	[103]

Where: 5637—human bladder carcinoma cells; A549—human lung carcinoma cells; ac.—acetone; AGS—human gastric adenocarcinoma cells; aq.—aqueous; B16—murine tumor cells; CD—cell death; Ctx = cytotoxicity; CV = cell viability; EC_50_—half maximal effective concentration; eth.—ethanol; H1299—human non-small cell lung carcinoma cells; HCepG-2—human hepatocellular carcinoma cells; HCT-116—colorectal cancer cells; HeLa—human cervical epithelioid carcinoma cells; HepG2—human hepatocyte carcinoma cells; HT-29—human colon cancer cells; LNCaP—prostate adenocarcinoma cells; MCF-7 breast adenocarcinoma cells; MDA-MB-231—Invasive ductal carcinoma cells (triple negative breast cancer); MDA-MB-453—human breast cancer cell line (non-triple negative breast cancer); MDA-MB 468—human breast cancer cells; meth.—methanol; MG-63—osteoblast cells; MTT—3-(4,5-dimethylthiazol-2-yl)-2,5-diphenyltetrazolium bromide; PANC-1—pancreatic cancer cells; RBC—red blood cells; SiHa—cervical cancer cells; TCT-116—human colon carcinoma cells; and WI 38—human lung fibroblast cells.

**Table 4:** Antitumoral potential of other phytosynthesized nanoparticles (as presented in studies published in 2019).

NPs	Extract Used	NPs Characteristics	Cell Lines	Main Findings	Ref.
ZnO NPs	*Tecoma castanifolia* (D.Don) Melch. leaves aq. extract	Spherical, 70–75 nm	A549	MTT assay—EC_50_ = 65 mg/L	[59]
ZnO NPs	*Pandanus odorifer* (Forssk.) Kuntze leaves aq. extract	Spherical, 90 nm	MCF7, HepG2, A549	MTT assay—CV < 65% after 24 h, at 100 mg/L	[61]
ZnO NPs	*Rhamnus virgate* Roxb. leaves aq. extract	Hexagonal, triangular, 20–30 nm	HepG2	MTT assay—EC_50_ = 19.67 mg/L	[63]
ZnO NPs	*Euphorbia heterophylla* L. leaves aq. Soxhlet extract	Hexagonal, 40 nm	A549, HepG2	MTT assay—EC_50_ = 383.05/329.67 mg/mL	[64]
ZnO NPs	*Allium cepa* L. leaves aq. extract	Hexagonal, cubic, 50 nm	A549	MTT assay—EC_50_ = 51.25 mg/L	[66]
ZnO NPs	*Hyssops officinalis* L. aq. extract	Pseudo-spherical, 20–40 nm	MDA-MB-231, MCF7	MTT assay—CV = 7/4% after 72 h at 500/100 mg/L	[104]
ZnO NPs	*Rheum turkestanicum* Janisch rhizome aq. extract	Spherical, 32.9 nm	WEHI 164	MTT assay—EC_50_ = 212.5 mg/L	[105]
ZnO NPs	*Scutellaria baicalensis* Georgi roots aq. extract	Spherical, 33.14–99.03 nm	HeLa	XTT assay—CV = 59.03% at 1000 mg/L	[106]
ZnO NPs	*Gracilaria edulis* (S.G.Gmelin) P.C.Silva aq. extract	Rod-shaped, 1.39 nm	SiHa	MTT assay—EC_50_ = 35 mg/L	[107]
ZnO NPs	*Annona squamosa* L. leaves aq. extract	Hexagonal, 20–50 nm	HeLa	MTT assay—EC_50_ = 50 mg/L	[108]
ZnO NPs	*Artocarpus heterophyllus* Lam. leaves aq. extract	Spherical, 12–24 nm	HCT-116	MTT assay—EC_50_ = 20 mg/L	[109]
Fe_2_O_3_ NPs	*Rhamnus virgate* Roxb. leaves aq. extract	Spherical, 20 nm	HepG2	MTT assay—EC_50_ = 13.47 mg/L	[68]
Fe_3_O_4_ NPs	*Cydonia oblonga* Miller seeds aq. Extract	Spherical, <50 nm	A549	MTT assay—CV approx. 40%, at 100 mg/L	[110]
Fe_2_O_3_, PbO NPs	*Papaver somniferum* L. pods aq. extract	Elliptical, spherical, 38 nm/Irregular, 23 nm	HepG2	SRB method—CV = 20.88%/38.49% after 24 h at 200 mg/L	[111]
Fe NPs	*Camellia sinensis* (L.) Kuntze leaves aq. extract	Spherical, 31.84 nm	SW1353	MTT assay—CV = 62% at 150 mg/L	[112]
CeO_2_ NPs	*Origanum majorana* L. leaves aq. extract	Spherical, 20 nm	MDA-MB-231	MTT assay—CV = 41.47% after 48 h at 125 mg/L	[113]
CeO_2_ NPs	*Ceratonia siliqua* L., 1753 leaves aq. extract	Spherical, 22 nm	MCF7	MTT assay—CV = 38.67% after 72 h at 1000 mg/L	[114]
CeO_2_ NPs	*Salvadora persica* L. bark aq. extract	Spherical, 10–15 nm	HT-29	MTT assay—CV = 80% after 24 h at 800 mg/L	[115]
CoO NPs	*Geranium wallichianum* Oliv. leaves aq. extract	21 nm	HepG2	MTT assay—EC_50_ = 31.4 mg/L	[73]
MgO NPs	*Sargassum wightii* Greville ex J.Agardh, 1848 aq. extract	Flower shaped, 68.02 nm	A549	MTT assay—EC_50_ = 37.5 mg/L	[70]
NiO NPs	*Abelmoschus esculentus*(L.) Moench leaves aq. extract	Spherical, 18.6 nm	Neuro2a	MTT assay—CV approx. 58% at 500 mg/L	[72]
Se NPs	*Spermacoce hispida* L. leaves aq. extract	Spherical, 50 nm	HepG2	MTT assay—CV = 50% at 30 mg/L	[116]
ZnS NPs	*Stevia rebaudiana* Bertoni leaves aq. Extract	Spherical, 8.35 nm	MCF7	MTT assay—EC_50_ = 400 mg/L	[117]
CuO, ZnO, CuO/ZnO NPs	*Alchornea cordifolia* Müll.Arg. leaves aq. extract	Spherical, star-like (for the composite), 16.25/75.22/3.54 nm	HeLa	MTT assay—CV = 63.64/44.05/39.94 after 48 h at 100 mg/L	[118]
Ag/TiO_2_ NPs	*Acacia nilotica* (L.) Wild. ex Delile leaves aq. extract	Spherical, 17 nm	MCF7	MTT assay—CV approx. 45% after 24 h at 100 μM	[74]
Fe_3_O_4_/Au NPs	*Juglans regia* L. husk aq. extract	Core-shell, 6.08 nm	HT-29	MTT assay—EC_50_ = 235 mg/L	[119]

Where: A549—human lung carcinoma cells; aq.—aqueous; CV = cell viability; EC_50_—half maximal effective concentration; H4IIE-luc—rat hepatocellular carcinoma; HCT-116—colorectal cancer cells; HeLa—human cervical epithelioid carcinoma cells; HepG2—human hepatocyte carcinoma cells; HT-29—human colon cancer cells; HuTu-80—human duodenal adenocarcinoma cells; MCF-7 breast adenocarcinoma cells; MDA-MB-231—Invasive ductal carcinoma cells (triple negative breast cancer); MTT—3-(4,5-dimethylthiazol-2-yl)-2,5-diphenyltetrazolium bromide; Neuro2a—fast-growing mouse neuroblastoma cells; SiHa—cervical cancer cells; SRB—sulforhodamine B; SW1353—human chondrosarcoma cells; WEHI 164—murine fibrosarcoma cells; and XTT—2,3-Bis(2-methoxy-4-nitro-5-sulfophenyl)-2H-tetrazolium-5-carboxanilide inner salt.

**Table 5:** Biocompatibility studies regarding phytosynthesized nanoparticles (as presented in studies published in 2019).

NPs	NPs Characteristics	Cell Lines	Main Findings	Ref.
Ag NPs	Spherical, 33.93 nm	VERO	MTT assay—EC_50_ = 12.5 mg/L	[18]
Ag NPs	Spherical, 35–55 nm	VERO	MTT assay—EC_50_ = 5.3 mg/L	[24]
Ag NPs	Spherical, 10–50 nm	HEK-293	MTT assay—EC_50_ = 23 mg/L	[26]
Ag NPs	Spherical, 22.7 nm	WI-38	CV = 90% at 100 mg/L	[27]
Ag NPs	Spherical, 5.76–19 nm	3T3	MTT assay—EC_50_ = 110 mg/L after 24 h	[29]
Ag NPs	Spherical 20–40 nm	HUVEC	MTT assay—EC_50_ = 760 mg/L	[45]
Ag NPs	Spherical, 8.83/11.24 nm	HaCaT	EC_50_ = 490/300 mg/L	[84]
Ag NPs	Spherical, 28.8 nm	HEK-293	MTT assay—EC_50_ = 1844 mg/L after 24 h	[88]
Ag NPs	Spherical, 24 nm	hPBLs	MTT—CV = 70% at 80 mg/L	[89]
Au NPs	Spherical, 10–60 nm	PBMC	MTT assay—EC_50_ = 600 mg/L	[53]
Au NPs	Spherical, triangular, hexagonal, 10–40 nm	HEK-293	WST-1 method, CV > 95% at 500 mg/L	[94]
Au NPs	Spherical, 2–10 nm	HEK-293	MTT assay—CV > 80% at 400 mg/L after 24 h	[101]
Au NPs	Spherical, triangular, hexagonal, 5–45 nm	MCF-10A	MTT assay—CV = not affected after 48 h at 200 mg/L	[103]
ZnO NPs	Hexagonal, triangular, 20–30 nm	RBC	MTT assay—EC_50_ > 200 mg/L	[63]
ZnO NPs	Hexagonal, 20–50 nm	HEK-293	MTT assay—CV = 76% at 200 mg/L	[108]
ZnO NPs	Spherical, 12–24 nm	VERO	MTT assay—EC_50_ = 30 mg/L	[109]
Fe_2_O_3_ NPs	Spherical, 20 nm	RBC	MTT assay—EC_50_ > 200 mg/L	[68]
Fe_2_O_3_, PbO NPs	Elliptical, spherical, 38 nm/Irregular, 23 nm	RBC	SRB method—CV = 59%/50.3% after 24 h at 400 mg/mL	[111]
Fe NPs	Spherical, 31.84 nm	VERO	MTT assay—CV = 80% at 150 mg/L	[112]
Se NPs	Spherical, 15–40 nm	N2a	MTT assay—EC_50_ = 127.28 mg/L	[58]
Se NPs	Spherical, 50 nm	VERO	MTT assay—CV not affected after 48 h at 60 mg/L	[116]
Cu NPs	Spherical, 45–50 nm	HUVEC	MTT assay—CV > 85% after 48 h at 1000 mg/L	[54]
CeO_2_ NPs	Spherical, 20 nm	HUVEC	MTT assay—CV = 87.67% after 72 h at 1000 mg/L	[113]
CeO_2_ NPs	Spherical, 23 nm	Lymphocytes	MTT assay—CV = 99.38% at 2.5 mg/L	[114]
CoO NPs	21 nm	Human macrophages and erythrocytes	MTT assay—EC_50_ > 200 mg/L	[73]
MgO NPs	Flower shaped, 68.02 nm	PBMC	MTT assay—CV > 95% after 24 h at 100 mg/L	[70]
Au, ZnO and Au/ZnO core-shell NPs	Spherical, 20–50 nm	Mouse fibroblast cells	MTT assay—CV = >80%/>50%/>70% at 250 mg/L	[78]
Fe_3_O_4_/Au NPs	Core-shell, 6.08 nm	3T3	MTT assay—EC_50_ > 500 mg/L	[119]

Where: 3T3—normal skin fibroblast cells; CV = cell viability; EC_50_—half maximal effective concentration; HaCaT—spontaneously transformed aneuploid immortal keratinocyte cell line from adult human skin; HEK-293—human embryonic kidney cells; hPBLs—human peripheral blood lymphocyte cultures; HUVEC—human umbilical vein endothelial cells; MCF-10A—non-tumorigenic epithelial cells; MTT—3-(4,5-dimethylthiazol-2-yl)-2,5-diphenyltetrazolium bromide; N2a—*Mus musculus* neuroblastoma cells; PBMC—peripheral blood mononuclear cells; RBC—red blood cells; SRB—sulforhodamine B; VERO—monkey kidney epithelial cells; WI-38—diploid human fibroblasts lung tissue cells.

**Table 6:** Toxicological studies regarding phytosynthesized nanoparticles (as presented in studies published in 2019).

NPs	Plant Material	NPs Characteristics	Test Organisms	Main Findings	Ref.
Ag NPs	*Selaginella bryopteris* leaves meth.: aq. (1:1) extract	Spherical, 5–10 nm	Mice injected with different doses (10–200 μg) of NPs	No hemorrhage and edema observed in experimental mice up to 100 μg	[20]
Ag NPs	*Allium sativum* L. aq. extract	Spherical, 10–50 nm	*Ceriodaphnia cornuta* G. O. Sars, 1885 (*Daphniidae*) exposed to 5–250 μg/L for 24 h	No mortality recorded at to 250 μg/L, affection of the swimming behavior at 250 μg/L (erratic swimming, migration to the bottom of the beaker or the water surface).	[26]
Ag NPs	*Piper longum* L. leaves aq. extract	Spherical, 28.8 nm	*Mesocyclops thermocyclopoides* Harada, 1931 (*Cyclopidae*) exposed to 250 solution for 72 h	No toxicity recorded	[88]
Ag NPs	*Elaeodendron croceum* (Thunb.) DC. stem bark aq. extract	Spherical, 12.62–41.44 nm	Acute oral toxicity evaluated on Wistar rats administered 500–2000 mg/kg NP doses	LD_50_ > 2000 mg/kg, no significant difference for mean organ-to-body weight ratio except in the liver and in all hematological parameters except WBC and hematocrit; no significant difference for serum electrolytes. total protein, urea, GGT, AST, ALP, ALT, albumin, bilirubin; changes in creatinine, urea, and cholesterol levels.	[91]
Ag NPs	*Solanum nigrum* L. leaves aq. extract	Spherical, 10–50 nm	*Ceriodaphnia cornuta*, *Paramecium* sp., *Poecilia reticulata* (guppy fish)	*C. cornuta*: LC_50_ = 23.5 mg/L, 100% lethality at 50 mg/L after 24 h, abnormal swimming behavior at lower concentrations; *Paramecium*: LC_50_ = 15.5 mg/L, 100% lethality at 30 mg/L after 5 min, morphological deformities (blackening, swelling, spindle shape deformity, blackening of cytoplasm) at lower concentrations; *fish*: LC_50_ = 38.3/34.5 mg/L after 48/96 h, 100% mortality at 50 mg/L after 96 h, no mortality under 20 mg/L., heart rate decreased with increasing concentration	[120]
Au NPs	*Halymenia dilatate* Zanardini aq. extract	Triangular, spherical, 16 nm	*Danio rerio* (F. Hamilton, 1822) (zebrafish) embryo exposed to 0–100 mg/L NPs for 96 h	No mortality or morphology variations after 96 h at 100 mg/L	[54]
Au NPs	*Cleome viscosa* L. leaves aq. extract	Spherical, 1–1.5 nm	Wistar male rats treated with 2, 5, 10 mg/kg released into the lungs	Increased amount of Au in serum and heart, LDH and CK-MB activities, cardiovascular injuries	[121]
ZnO NPs	*Rhamnus virgate* Roxb. leaves aq. extract	Hexagonal, triangular, 20–30 nm	*Artemia* sp. (brine shrimps) exposed to 1–200 mg/L NPs for 24 h	LC_50_ = 26.34 mg/L	[63]
Zn NPs	*Lavandula vera* DC. leaves aq. extract	Spherical, 30–80 nm	Oral acute and subacute toxicity in male NMRI mice administered NPs by oral gavage for 14 days	LC_50_ = 5.5 g/kg (non-toxic); low oral toxicity at 1, 2 and 3 g/kg after 14 days; sub-acute effects—changes in the body weight, hematological parameters, no toxicological effects at 1 g/kg	[122]
Fe_2_O_3_ NPs	*Rhamnus virgate* Roxb. leaves aq. extract	Spherical, 20 nm	*Artemia* sp. (brine shrimps) exposed to 1–200 mg/L NPs for 24 h	LC_50_ = 32.41 mg/L	[68]
CoO NPs	*Geranium wallichianum* Oliv. leaves aq. extract	21 nm	*Artemia* sp. (brine shrimps) exposed to 1–200 mg/L NPs for 24 h	LC_50_ = 18.12 mg/L	[73]
CeO_2_ NPs	*Rhus punjabensis* J. L. Stewart ex Brandis stem aq. extract	Spherical, 23 nm	Female Sprague-Dawley rats orally administered doses of 200/400 mg/kg body weight for 14 days	No effect on serum biochemistry, except for creatine phosphokinase (significantly reduced)	[114]

Where: ALP—alkaline phosphatase; ALT—alanine aminotransferase; aq.—aqueous; AST—aspartate aminotransferase; CK-MB—creatine kinase-MB isoenzyme; GCT—ɣ-glutamyl transferase; LC_50_—half maximal lethal concentration; LDH—serum lactate dehydrogenase; meth.—methanol; N2a—*Mus musculus* neuroblastoma cells; WBC—white blood cells.
